# Understanding the Impact of Infection, Inflammation, and Their Persistence in the Pathogenesis of Bronchopulmonary Dysplasia

**DOI:** 10.3389/fmed.2015.00090

**Published:** 2015-12-21

**Authors:** Jherna Balany, Vineet Bhandari

**Affiliations:** ^1^Section of Neonatology, Department of Pediatrics, St. Christopher’s Hospital for Children, Drexel University College of Medicine, Philadelphia, PA, USA

**Keywords:** premature newborn, chronic lung disease, cytokines, sepsis, hyperoxia, mechanical ventilation

## Abstract

The concerted interaction of genetic and environmental factors acts on the preterm human immature lung with inflammation being the common denominator leading to the multifactorial origin of the most common chronic lung disease in infants – ­bronchopulmonary dysplasia (BPD). Adverse perinatal exposure to infection/inflammation with added insults like invasive mecha nical ventilation, exposure to hyperoxia, and sepsis causes persistent immune dysregulation. In this review article, we have attempted to analyze and consolidate current knowledge about the role played by persistent prenatal and postnatal inflammation in the pathogenesis of BPD. While some parameters of the early inflammatory response (neutrophils, cytokines, etc.) may not be detectable after days to weeks of exposure to noxious stimuli, they have already initiated the signaling pathways of the inflammatory process/immune cascade and have affected permanent defects structurally and functionally in the BPD lungs. Hence, translational research aimed at prevention/amelioration of BPD needs to focus on dampening the inflammatory response at an early stage to prevent the cascade of events leading to lung injury with impaired healing resulting in the pathologic pulmonary phenotype of alveolar simplification and dysregulated vascularization characteristic of BPD.

## Introduction

Bronchopulmonary dysplasia (BPD) is the most common chronic respiratory disease affecting infants wherein the developmental program of the lung is altered secondary to preterm birth of the baby (1). Lung development progresses in five distinct stages: embryonic, pseudoglandular, canalicular, saccular, and alveolar (2, 3). Human preterm babies who develop BPD are born in the late canalicular or early saccular stage of lung development. The late canalicular stage is characterized by development of the primitive alveoli and the alveolar capillary barrier, and the differentiation of type I and type II pneumocytes. The early saccular stage is marked by initiation of surfactant production, pulmonary vascularization, and enlargement of terminal airways (2–5). Unique to lung development is the fact that unlike other organs, the lungs complete their development after birth (up to 8 years of age) (6). Alveolar sacs are formed by secondary septation of alveolar ducts. With preterm birth, this programed development is disrupted, and in the setting of inflammation [whether it is due to infection, mechanical ventilation (MV), or hyperoxia] causes impaired alveolarization leading to BPD. We need to remember that while in sheep, baboons, and humans, the saccular stage occurs *in utero*; in rodent models, it begins at embryonic day 18 and continues through postnatal (PN) day 5 (4, 5).

In spite of many advances in neonatal medicine in the past few decades, like the introduction of better MV strategies and the use of surfactant and antenatal steroids, the incidence of BPD has not declined (7). The incidence of BPD in the United States is about 10,000–15,000 new cases each year out of which the majority of those affected have a birth weight <1250 g (8). Pulmonary and neurodevelopmental sequelae of this devastating disease extend even into adulthood (9).

**Figure 1:**
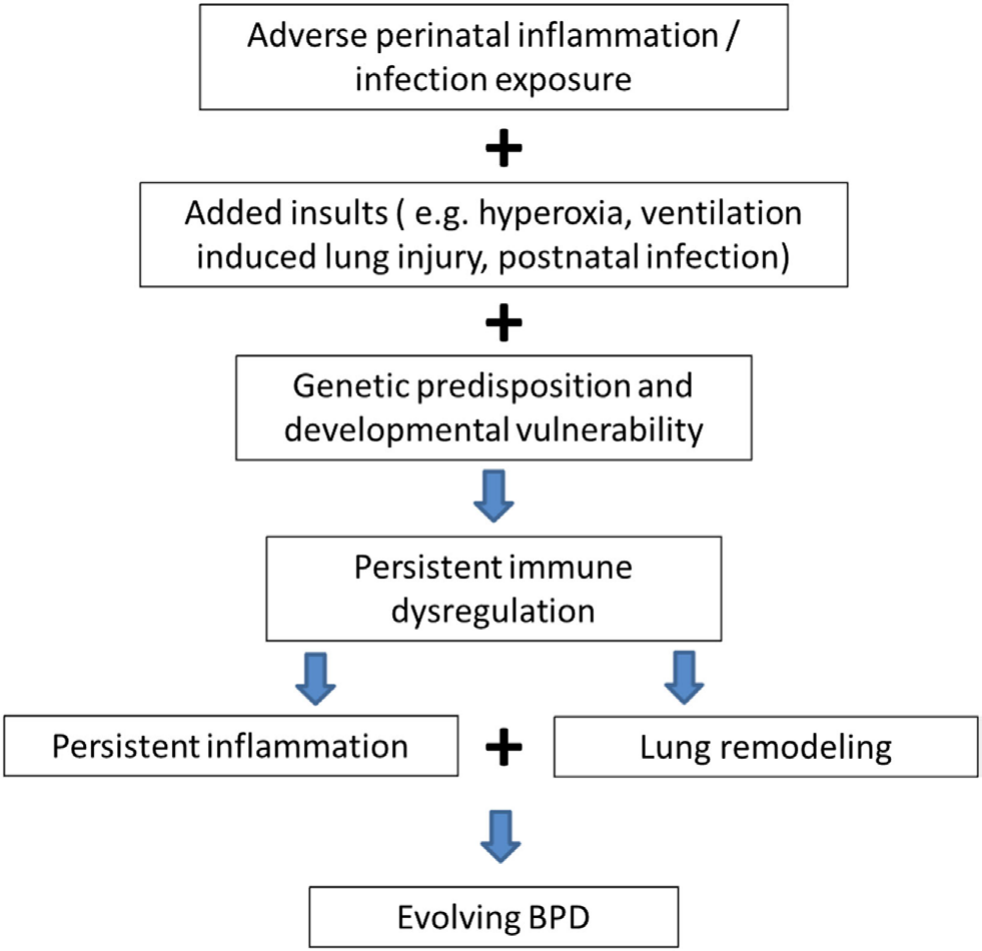
**Genetic predisposition and persistent inflammation due to environmental factors (sepsis, invasive mechanical ventilation, and hyperoxia) acting on the foundation of immature lung underlie the pathogenesis of BPD**.

Genetic (10) and environmental factors (pre- and/or postnatal sepsis, invasive MV, and hyperoxia) (1) act on the preterm human immature lung with inflammation being the common denominator in all these interactions leading to the multifactorial origins of this disease. As shown in Figure 1, it is postulated that adverse perinatal exposure/infection with added insults like invasive MV, exposure to hyperoxia, and sepsis causes persistent immune dysregulation. This on top of genetic susceptibility and prematurity leads to persistent inflammation leading to lung remodeling and evolution of BPD.

In this review article, we have attempted to analyze and consolidate current knowledge about the role played by persistent prenatal and postnatal inflammation in the pathogenesis of BPD. We searched PubMed for articles limited to English language with the keywords: “Bronchopulmonary dysplasia or BPD,” “inflammation,” “chorioamnionitis,” “mechanical ventilation,” “hyperoxia,” “postnatal sepsis,” either individually or in combination. We focused on articles published over the last 10 years and used the most relevant ones for this review.

## Mediators of Inflammation in BPD

Bronchopulmonary dysplasia has been linked to the development of an inflammatory response that can occur in absence of clinical infection. Systemic fetal inflammatory response (11) and neonatal leukemoid reactions (12) have been implicated as a risk factor for BPD. Pulmonary inflammation in BPD is characterized by the presence of inflammatory cells like neutrophils and monocytes, pro-inflammatory cytokines, and other mediators, including soluble adhesion molecules.

The innate immunity and adaptive immunity reinforce each other and act in unison. Cells of the innate immune system secrete cytokines, which can prime lymphocytes thereby modulating adaptive immunity (13). Exposure to a specific antigen causes these primed lymphocytes to have a more rapid and intense immune response (14, 15). Naïve T cells express CD62L (L-selectin) (16). Upon activation, the T cells shed their surface CD62L molecules. In infants with BPD, the expression of the CD62L is decreased on these CD4+ T-cells thereby suggesting T cell activation. CD54 (intercellular adhesion molecule-1 or ICAM-1) is an adhesion molecule that mediates a co-stimulatory signal in T cell activation. CD54 expression is increased upon cell activation (17).

The premature lung is exposed to ongoing oxidative and cellular damage. Damaged lung tissue releases chemotactic factors and inflammatory cytokines, such as interleukin (IL)-1, IL-8 (CXCL-8), and tumor necrosis factor alpha (TNF-α). This leads to an influx of neutrophils and other inflammatory cells with increased release/production of additional pro-inflammatory cytokines (Table 1). Multiple studies have shown that IL-1β, IL-6, and IL-8 are elevated very early in the respiratory course of the human preterm population that ultimately develop BPD, and in tracheal aspirates of those with BPD. In contrast, decreased levels of IL-10 in serum and tracheal aspirates have been shown in studies of those infants who developed BPD. In addition to ILs, a large variety of other biomarkers have been detected and associated with the development of BPD in tracheal aspirates, as well as blood and urine samples of premature infants (9, 18). The ones that have been implicated in the animal models include inflammatory cytokines, matrix proteins, growth factors, and vascular factors (9, 18–33). Their role is illustrated in Table 1.

**Table 1:** **Selected mediators of inflammation, their role, and corresponding expression in BPD**.

Mediators of inflammation	Role	Expression in BPD
**Inflammatory cytokines**		
Interleukins: anti-inflammatory		
IL-10	Suppresses inflammatory response by inhibiting NF-κB	↓/↔
IL-4, IL-13	Suppresses inflammation by inhibiting pro-inflammatory cytokine production	↔
Interleukins: pro-inflammatory		
IL-1, IL-6	Acute phase inflammatory response	↑
IL-8 (CXCL-8)	Main chemoattractant for neutrophils	↑
CC chemokines		
Monocyte chemoattractant protein (MCP)-1, 1α, 1β, 2, 3	Recruit inflammatory cells to area of injury	↑
Macrophage migration inhibitory factor (MIF)	Upstream regulator of innate immune response	↓
Tumor necrosis factor alpha (TNF-α)	Enhances expression of other pro-inflammatory cytokines	↑
Transforming growth factor-beta 1 (TGF-β1)	Pro-inflammatory	↑
**Matrix proteins**		
Matrix metalloproteinase-8	Disordered pulmonary remodeling after inflammation	↑
Matrix metalloproteinase-9	Pro-inflammatory, interferon-gamma (IFN-γ) signaling	↑
**Growth factors**		
Endothelin-1	Pro-inflammatory	↑
Vascular endothelial growth factor	Pro-inflammatory	↑/↓
Connective tissue growth factor (CTGF)	Pro-inflammatory	↑
Bombesin-like peptide (BLP)	Increases mast cells in the lung	↑
Breast regression protein-39 (human analog is YKL-40)	Anti-inflammatory	↓
Pulmonary hepatocyte growth factor (HGF)	Alveolar septation, repair	↓
Keratinocyte growth factor (KGF)	Regulates proliferation of alveolar epithelial cells	↓
**Miscellaneous**		
Interferon-inducible protein 9 (IP-9 – also known as CXCL11)	Pro-inflammatory, IFN-γ signaling	↑
Cyclooxygenase-2 (Cox-2)	Pro-inflammatory, IFN-γ signaling	↑
CCAAT/enhancer-binding protein (C/EBP)	Pro-inflammatory, IFN-γ signaling	↑
Endoglin	Pro-inflammatory	↑
Periostin	Pro-inflammatory	↑
Clara cell secretory protein	Modulates acute pulmonary inflammation	↓
Parathyroid hormone-related protein (PTHrP)	Alveolar growth	↓
Angiopoietin-2	Pro-inflammatory	↑
Lactoferrin	Anti-inflammatory	↓

*↑ – increase; ↔ – no change; ↓ – decrease*.

We will now describe the major environmental factors that contribute to inflammation, and its persistence, in the pathogenesis of BPD. These include prenatal influences (chorioamnionitis) and postnatal influences, namely early- and late- onset sepsis, invasive MV, and hyperoxia.

## Prenatal Factors Causing Inflammation – Chorioamnionitis

As the name suggests, chorioamnionitis is inflammation of the chorion and amnion membranes of the placenta (34). Although commonly seen in clinical practice, chorioamnionitis is a ­complex syndrome associated with pregnancy leading to preterm deliveries (34). Chorioamnionitis has been classified as either histological or clinical. With histological chorioamnionitis, there is infiltration of polymorphonuclear leukocytes and other inflammatory cells like macrophages and T cells as seen microscopically (35–37). Clinical chorioamnionitis is evidenced by fever >37.5°C, uterine tenderness, foul smelling vaginal discharge, abdominal pain, maternal tachycardia with a heart rate >100 bpm, fetal ­tachycardia HR >160 bpm, and white blood cell (WBC) count >15,000/mm^3^ (38, 39).

It has been shown in *in vitro* studies that bacterial products like phospholipase A2, peptidoglycan polysaccharide, proteolytic enzymes, and endotoxins can initiate an inflammatory response. Inoculation of the amniotic cavity with *E. coli* lipopolysaccharide (LPS) or live *Ureaplasma* organisms has been shown to induce structural and functional fetal lung maturation (40–43). Antenatal lung inflammation impacts a variety of signaling pathway regulators like toll-like receptors 2 and 4 (TLR2 and TLR4), growth factors like TGF-β and CTGF, and mesenchymal structural proteins like bone morphogenetic protein-4 leading to vascular remodeling and alveolar simplification, which could be considered akin to a mild BPD phenotype (40–43). However, repetitive LPS exposure and/or chronic chorioamnionitis leads to immune tolerance and a dampened inflammatory response, which in turn allows the lungs to develop close to normal in experimental BPD animals (40–42).

Adverse perinatal outcomes are seen with intra-amniotic inflammation irrespective of the presence of intra-amniotic infection. Colonization *per se*, without inflammation is not associated with adverse outcomes (44). The severity of the adverse outcomes is directly related to the severity of the intra-amniotic inflammation (44). Maternal antibiotic use has been associated with decreased BPD (45).

To summarize, in experimental animals, antenatal inflammation causes lung maturation and some degrees of lung injury, which is modified by the not fully developed innate immune response, exposure to antenatal steroids, and noxious postnatal factors. Not surprisingly, given the variability in definition and impact of various confounding factors, the issue of antenatal inflammation causing BPD in human infants is controversial (42, 46–49). Chorioamnionitis increases the incidence of preterm birth, and if accompanied by lung inflammation could result in surfactant dysfunction allowing for prolonged exposure to supplemental oxygen and invasive MV (11, 48). This “multiple hit” of events could explain the propensity to BPD in such infants (48), though this has not been consistently shown (50). In addition, persistence and non-resolution of lung inflammation lead to BPD by inhibiting secondary septation, alveolarization and normal vascular development, and the compromised ability of the lungs to heal.

## Postnatal Factors Causing Inflammation – Sepsis

Preterm infants are more susceptible to infections since their immune defenses are not fully developed, have vulnerable skin barrier, and require multiple invasive procedures (51). Postnatal infection/inflammation could either be localized to the lung or could be systemic in origin. Chorioamnionitis increases the risk of early-onset neonatal sepsis, which sets off an inflammatory cascade (48). Also, it has been shown that late-onset sepsis induces a pro-inflammatory and pro-fibrotic response in the preterm lung predisposing it to BPD (51).

Local (intra-tracheal) exposure to LPS (bacterial endotoxin) or dsRNA (a marker of viral replication) in the neonatal rat led to acute cellular and cytokine inflammatory responses, which were associated with histologic features of impaired alveolar development (52, 53).

Neonatal mice injected with intraperitoneal LPS demonstrated reduced lung inflammation and apoptosis after 24 h as compared to adults, and this was associated with activation of the transcription factor, nuclear factor kappa B (NF-κB) (54). Inhibition of NF-κB resulted in increased cell death and alveolar simplification and disruption of angiogenesis via vascular growth factor (VEGF)-R2 (55). It has also been shown that using a targeted deletion of NF-κB signaling (using a lung epithelium-specific deletion of IKKβ – which is a known activating kinase upstream of NF-κB) in a mouse model results in alveolar hypoplasia with decreased VEGF expression (56). In addition, there was increased expression of CXCL-1, as well as its receptor CXCR2. Pre-treatment with CXCR2-neutralizing antibody was able to reverse the effects in the developing lung (53). In summary, exposure to either bacterial or viral agents in the rodent model led to features of inflammation, with pulmonary histology suggestive of BPD.

Inflammatory response secondary to viral infections in early post natal stages could be worth considering in the evolution of BPD. Increased neutrophil accumulation, increased expression of CXCL-1 and its receptor CXCR2, and decreased lung alveolarization have been seen with intra-tracheal delivery of viral pro-inflammatory dsRNA in 10-day-old mouse model (53).

## Postnatal Factors Causing Inflammation – Invasive Mechanical Ventilation

Mechanical ventilation is a risk factor for the development of BPD in premature infants. Lung injury from MV results due to volutrauma, barotrauma, or atelectrauma (57).

When lungs are exposed to high tidal volumes, over distension leads to production of pro-inflammatory cytokines like IL-6, IL-8, and TNFα and reduced expression of anti-inflammatory cytokines like IL-10 (58). Even ventilation at low tidal volumes is deleterious because of the stretch injury it can induce by over-distending partially collapsed lungs. Sustained lung inflation (SLI) has been shown to increase levels of pro-inflammatory cytokines and BPD-like changes in the lungs of preterm lambs (59). There is great need to find non-invasive ventilation strategies for preterm neonates because even “gentle” invasive MV for a shorter duration can induce an inflammatory response (60).

In neonatal rats (7- to 14-day-old – in the alveolar phase of lung development), high tidal volume ventilation increased IL-6 mRNA and upregulated the TGF-β signaling molecule, CTGF mRNA, and protein expression compared to controls (61). In an 8-day-old rat ventilation model, high tidal volumes increased the neutrophilic and inflammatory cytokine mRNA and/or protein expression (IL-1β, IL-6, CXCL-1 and 2) response (62). In a 7-day-old rat model, exposure to MV for 24 h in room air led to cell cycle arrest (63), suggesting a harbinger to alveolar simplification, the pathologic hallmark of BPD.

In an invasive MV model in 2-week-old mice (well into the alveolar phase of lung development) for 1 h, IL-6 lung levels were increased in the high tidal volume ventilation group (64). Studies conducted in 2- to 6-day-old mice (late saccular to early alveolar phase of lung development) ventilated for 8–24 h with room air or 40% O_2_ revealed dysregulated elastin (ELN) assembly, a threefold to fivefold increase in cell death, TGF-β activation, and a decrease in VEGF-R2 expression (65, 66). Inhibiting lung elastase activity by using recombinant human elafin or genetically modified mice that expressed elafin in the vascular endothelium was protective of the lung injury (67, 68).

Early studies using a chronically ventilated (3–4 weeks) preterm lamb model of BPD showed evidence of non-uniform inflation patterns and impaired alveolar formation with an abnormal abundance of elastin (69). Inflammation was evident by the presence of inflammatory cells, namely alveolar macrophages, neutrophils, and mononuclear cells and edema (69). In this model, there was also reduced lung expression of growth factors that regulate alveolarization and differential alteration of matrix proteins that regulate ELN assembly (70). A non-invasive (nasal) ventilation approach preserved alveolar architecture (71) and had a positive effect on parathyroid hormone-related protein-peroxisome proliferator-activated receptor-gamma (PTHrP-PPARγ)-driven alveolar homeostatic epithelial–mesenchymal signaling in the preterm lamb model (72).

It has been seen in preterm fetal sheep that there is increased expression of early response gene-1 (Egr-1) as well as pro- and anti-inflammatory cytokines and dynamic changes in heat shock protein 70 (HSP70) (57). This stretch injury also increases expression of granulocyte/macrophage colony-stimulating factor mRNA leading to maturation of lung monocytes to alveolar macrophages (57). Induction of surfactant proteins A, B, and C mRNA is also increased (57). More recently, even short-term stretch injury (15 min) secondary to invasive MV in preterm fetal sheep led to increased levels of pro-inflammatory cytokines, IL-1β, IL-6, monocyte chemoattractant protein (MCP)-1, and MCP-2 mRNA by 1 h (57). This was accompanied by increased presence of inflammatory cells in the bronchoalveolar lavage fluid (BALF) with initial increases in neutrophils and monocytes by 1 h and a transition to macrophages by 24 h (57).

The preterm ventilated baboon model of BPD (delivered at 125 days – at 68% of gestation) showed evidence of alveolar hypoplasia and dysmorphic vasculature, akin to that seen in human BPD (73). Importantly, there were significant elevations of TNF-α, IL-6, IL-8 levels, but not of IL-1β and IL-10, in tracheal aspirate fluids at various times during the period of ventilator support, supporting a role for inflammation (73). In addition, increased matrix metalloproteinase-9 (MMP-9) levels were associated with lung inflammation and edema seen in this invasive ventilation model (74). Alteration of VEGF was also noted in the lungs of various baboon models (75, 76). Bombesin is a 14-amino acid peptide, initially detected in amphibian skin, but immunoreactive studies have shown the presence of bombesin-like peptide (BLP) in multiple organ systems in mammals (77). In the lung, BLP have been shown to be released by pulmonary neuroendocrine cells (77). BLP blockade improved alveolar septation and angiogenesis in the preterm baboon models (78, 79).

In the 125-day baboon model, treatment with early nasal continuous positive airway pressure (NCPAP) for 28 days led to a pulmonary phenotype similar to 156 days gestational control lungs, suggesting that this non-invasive approach could minimize lung injury (80). In the same model, delayed extubation (till 5 days) versus early extubation to NCPAP at 24 h led to significantly increased BALF IL-6, IL-8, MCP-1, macrophage inflammatory protein-1 alpha (MIP-1α), and growth-regulated oncogene-alpha (GRO-α) in the delayed NCPAP group (81).

Some epidemiological studies showed that replacing invasive MV with NCPAP was associated with BPD reduction (82). No reduction in the incidence of BPD or mortality in the NCPAP group was seen in the COIN study that randomized infants born at 25–28 weeks to receive either NCPAP or intubation with MV in the delivery room (83). The INSURE (IN: intubation, SUR: surfactant, E: extubation) technique has been shown to reduce the need for MV and incidence of BPD (84). Non-invasive ventilation strategies like nasal intermittent-positive pressure ventilation (NIPPV) not only reduce the need for intubation within the first 48–72 h of life, but also have been associated with decreased mortality and/or BPD and hence is a feasible option for the newborn (85–87), though additional studies are required (88). The optimal mode of non-invasive ventilation (for example: type of NCPAP, maximum level of NCPAP, synchronized or non-synchronized method of nasal ventilation), selection of the best nasal interface (short-prongs or mask), and choice of ventilator need to be determined, and this information would be helpful in management of the disease.

To summarize, while the lamb/sheep/baboon ventilation models are in the saccular stage (akin to the human premature babies who are at most risk for BPD at birth), the rat/mouse ventilation models are in the alveolar phase of lung development. However, it is quite obvious that mechanical stretch injury generates an inflammatory response (mostly neutrophils, IL-1β, IL-6, CXCL-1/-2, TGF-β signaling), along with alterations in matrix proteins (ELN, MMP-9) and VEGF. In addition, there is increased cell death and cell cycle arrest. Thus, it appears that an initial inflammatory cascade triggers the signaling of additional molecular mediators that lead to dysregulated vascularization and impaired alveolarization. Interestingly, non-invasive (nasal) ventilation approaches were protective of these responses. Thus, prolonged invasive MV sets off a persistent cascade of inflammatory response that in the setting of hyperoxia takes the “multiple hit” pathway of leading to BPD.

## Postnatal Factors Causing Inflammation – Hyperoxia

**Figure 2:**
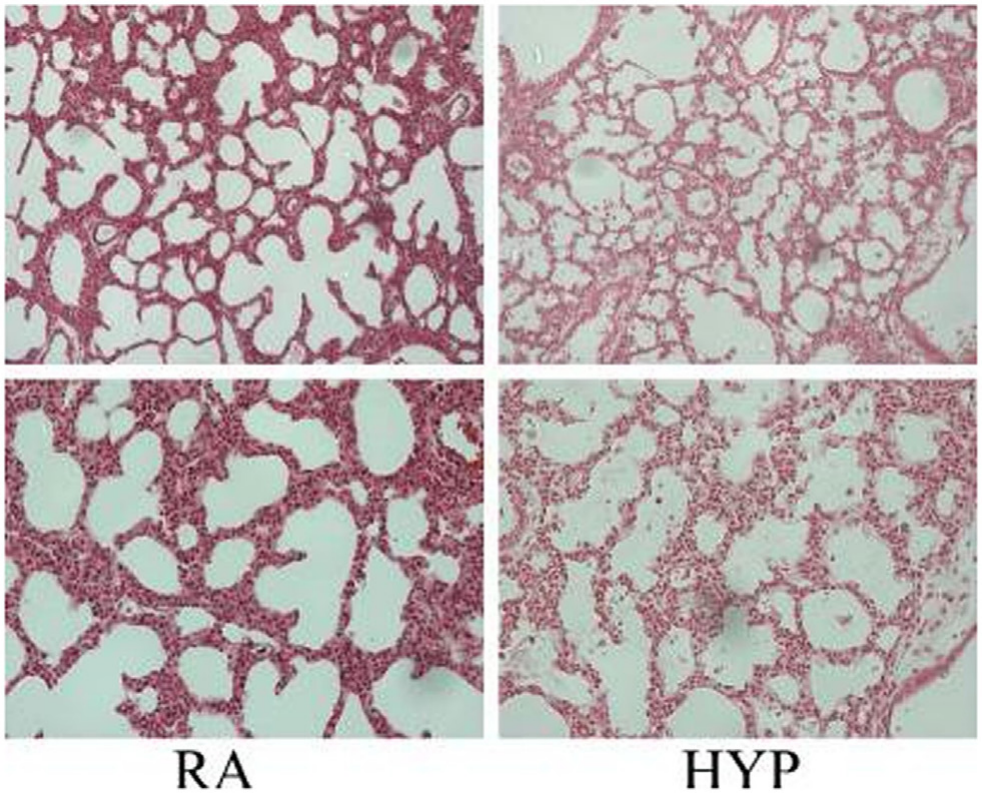
**Photomicrographs (×10, upper panel; ×20 lower panel; hematoxylin and eosin stain) of neonatal lung injury noted in newborn mice at postnatal day 2, after 100% O_2_ exposure since birth**. Note the alveolar exudates and presence of inflammatory cells in the hyperoxia-exposed lungs compared with litter-mate controls in room air. RA, room air; HYP, hyperoxia [with permission from *Semin Fetal Neonatal Med* (2010) 15(4):223–9].

Many studies have documented the injurious effects of perinatal supplemental oxygen on lung development. Target levels of O_2_ in extremely low birth weight (ELBW) have been studied extensively. The morphologic changes of human BPD resemble hyperoxic lung injury in newborn animals (73). Prolonged exposure to hyperoxia in the neonatal mouse for 14 days or longer results in a phenotype of “old” BPD (89, 90). Exposure to hyperoxia in the critical saccular stage of lung development replicates human BPD, with effects that are dose-dependent on the fraction of inspired oxygen (FiO_2_) concentration; the effects last lifelong with increased susceptibility to respiratory tract infections (91–95). Acute lung injury caused by hyperoxia (Figure 2) occurs secondary to an inflammatory response, which causes destruction of the alveolar–capillary barrier, vascular leak, influx of inflammatory mediators, pulmonary edema, and ultimately cell death (96). With continued exposure to hyperoxia this inflammatory response and pulmonary edema improve initially but chronic pulmonary inflammation ensues in the following weeks (97). At the cellular level, alveolar or interstitial macrophages express early response cytokines when exposed to hyperoxia, which in turn attract inflammatory cells to the lungs (19).

It has been shown that there exists a dose-dependent effect of hyperoxia on severity of BPD in the murine model. Mice exposed to varying concentrations of oxygen ranging from 40 to 100% at PN days 1–4 had more severe disease at higher concentrations of oxygen (92). An oxygen dose-dependent inflammatory response to influenza-A viral infection in adult mice that had been exposed to hyperoxia as neonates has been reported (95). Furthermore, this response was dependent upon the cumulative exposure to oxygen (98).

The specific role of individual inflammatory molecular mediators in the pathogenesis of BPD has been particularly well illustrated by utilizing lung-targeted overexpressing transgenic models, in room air, resulting in pulmonary phenotypes reminiscent of human BPD. These include IL-1β (99, 100) and IFN-γ (25, 91). In the case of IL-1β transgenic mice, absence of the beta6 integrin subunit was protective of the BPD phenotype (101). Interestingly, inhibition of cyclooxygenase-2 (Cox-2) ameliorated the BPD phenotype in the hyperoxia-induced as well as the IFN-γ lung overexpressing transgenic mouse model in room air. A recent paper has reported that increased Cox-2 activity may contribute to proinflammatory responses in hyperoxia-exposed developing mouse lungs (102).

There is increased expression of IL-1α mRNA in neonatal mice exposed to hyperoxia (89). Lung mRNA for IL-1β also increases in neonatal mice exposed to hyperoxia (103). Transgenic IL-1β overexpression in lung epithelium resulted in BPD phenotype in neonatal mice (100). In hyperoxia-exposed newborn rabbits, the pattern of IL-1β rise and fall matches the rise and fall of histologic inflammation (104). However, in the immature baboon model of BPD, no such pattern between IL-1β levels and inflammation was seen in the tracheal aspirates (73). CINC-1 in premature rat lungs (105) and newborn rabbits (104) exposed to hyperoxia was upregulated. Also, IL-8 levels in tracheal aspirates of the premature baboon model of BPD have been shown to be increased (73).

The lungs of hyperoxia-exposed neonatal mice had no change in IL-10 mRNA expression (103). Also tracheal aspirates of baboon model of BPD show no difference in IL-10 levels (73). IL-1β, IL-6, and IL-8 are pro-inflammatory cytokines and are elevated very early in the course of BPD.

Typically viewed as pro-inflammatory, these cytokines have been shown to be elevated very early in the respiratory course of the human preterm population that ultimately develops BPD (20). Studies have found that serum and tracheal aspirate IL-10 levels were decreased in those infants who developed BPD (20).

A variety of potential therapeutic agents have been used in hyperoxia-exposed mice models that have been shown to decrease inflammation and/or attenuate other parameters of lung injury/BPD phenotype. These include rosiglitazone (106, 107), hepatocyte growth factor (HGF) (108), B-naphthoflavone (109), arginyl-glutamine as well as docosahexaenoic acid (110), and a combination of vitamin A and retinoic acid (111). Treatment with human amnion epithelial cells attenuated some parameters of hyperoxia-induced inflammatory lung injury (mRNA expression of IL-1α, IL-6, TGF-β, platelet-derived growth factor-beta or PDGF-β, mean linear intercept, and septal crest density), but not other aspects, for example, alveolar airspace volume, collagen content, or leukocyte infiltration in neonatal mice (112).

To summarize, while variable initiation and duration of exposure to hyperoxia animal models have been reported as models of human BPD, exposure to hyperoxia for a relatively short (PN1–4) duration in mice, which is at the critical saccular stage of lung development, can result in an inflammatory response sufficient to create the BPD pulmonary phenotype. This can be recapitulated using transgenic mice models of the inflammatory mediators, but kept in room air. Importantly, exposure to 0.4, 0.6, >0.8 FiO_2_ can mimic mild, moderate, and severe BPD, respectively. A vast array of therapeutic agents has been reported to be effective in improving alveolar and/or vascular architecture of the hyperoxia-exposed neonatal lung in lambs, rats, and mice.

While hyperoxia exposure is a good starting point for testing the efficacy of potential therapeutic agents, it is important to be able to delineate the responsible molecule/signaling pathway in developmentally appropriate room air models and confirm the results in preventing/ameliorating the BPD phenotype. This would avoid the confounding variable of hyperoxia-induced alterations in multiple other molecular mediators, allowing delineation of targeted molecules in specific signaling pathways for maximal potential therapeutic relevance. Among the inflammatory mediators of hyperoxia-induced lung injury that can mimic the BPD phenotype in room air, the well-defined ones are IL-1β, TGF-β1, CTGF, IFN-γ, and MIF. It would be important to attempt to translate some of the newer targets in specific signaling pathways that have been recently reported, for example, inhibition of Cox-2 (91, 102) as a potential therapeutic option for prevention/amelioration of BPD.

## Persistent Inflammation in BPD

It is important to highlight the fact that for BPD to occur, it requires the known environmental factors to be exposed to the immature lung for a sustained duration, resulting in persistent inflammation. For the chorioamnionitis rodent models, the exposure to LPS is over a few days in the late canalicular/early saccular stage of lung development. For the relative short duration of exposure to invasive MV and hyperoxia in rodent models, 1 postnatal day in the saccular stage of lung development is equivalent to 3–4 weeks in a human preterm infant. Obviously, the larger animal models (sheep/lamb/baboon) also need few days to weeks of injury to develop the pulmonary phenotype of BPD. While some parameters of the early inflammatory response (neutrophils, cytokines such as IL-1, TNFα) may not be detectable after days to weeks of exposure to noxious stimuli, they have already initiated the signaling pathways of the inflammatory process/immune cascade and have affected permanent defects structurally and functionally in the BPD lungs. This is borne out by the facts that the pathologic appearance of large simplified alveoli is permanent following just the first 4 PN days of hyperoxia exposure in mice models (93). Furthermore, these mice have increased mortality when exposed to viral infectious challenge as adults (98, 113). In concordance, preterm neonates with BPD have anatomical and functional pulmonary deficits well into childhood and as adults (114–116). There is some clinical evidence that early interruption of the initial inflammatory response could result in amelioration and potential reversal of these effects (117).

## Summary and Conclusion

It is important to remember that while *in vitro* studies are helpful in figuring out the mechanistic significance of a signaling pathway, these are usually conducted with cell lines or freshly isolated single cells of a particular phenotype. Thus, the results of such studies may not accurately reflect the *in vivo* situation of interaction with the multiple cell types found in the lung. In addition, while the significantly different responses between adult and neonatal lungs to the postnatal factors discussed here – invasive ventilation (118–121), local/systemic sepsis (52, 54, 122–124), and hyperoxia (19, 125–127) – are well established, it is also important to be cognizant of the stages of lung development when comparing animal data for relevance to humans. This is best exemplified by studies that highlight the differential responses in the various stages of lung development (mostly, saccular vs. alveolar) in animal models (99). Furthermore, the degree and duration of exposure to the noxious stimulus (hyperoxia, for example) in the animal models needs to be appropriate in order to attempt to extrapolate the data to humans. For example, a prolonged exposure to hyperoxia from birth to 2 weeks in the mouse, i.e., almost to the end of alveolarization is akin to exposing a preterm neonate to the same to at least up to 2 years of age.

To conclude, it is the preterm lung in the late canalicular/saccular phase of development that is most predisposed to BPD, when exposed to the pre- and postnatal factors. Inflammation and then its persistence in the preterm lung – whether initiated by prenatal factors like chorioamnionitis or whether propagated postnatally with the use of high FiO_2_ and invasive MV or sepsis – culminates in BPD. Hence, translational research needs to be aimed at decreasing chorioamnionitis and finding better strategies for early non-invasive MV and optimum use of oxygen for the immature preterm lung for dampening the inflammatory response.

## Author Contributions

JB wrote the initial draft. VB did substantial re-organization and editing of the manuscript. Both authors have approved the final version of the manuscript as submitted.

## Conflict of Interest Statement

The authors declare that the research was conducted in the absence of any commercial or financial relationships that could be construed as a potential conflict of interest.
